# The International Medical Annual and Practitioner’s Index

**Published:** 1897-05

**Authors:** 


THE INTERNATIONAL MEDICAL ANNUAL AND PRACTITIONER'S INDEX. A Work of Reference for Medical Practitioners. Fifteenth year. New York: E. B. Treat, 241–243 W. 23d street. 1897.

This annual for 1897 comes with its usual amount of valuable material condensed and so arranged as to be easily consulted by the busy practitioner. For fifteen years the annual has held its own with many competitors, and the 1897 edition is a worthy successor in the long row of successful year-books.

The arrangement of the material does not differ from other years, considerable space being devoted to new remedies under the authorship of William Murrell, of Westminster hospital. Particularly interesting are the various reports on the serum preparations, all in all showing distinct advances, which insures their permanent position in therapeutics. The review of new treatment, under the able staff of contributors, is exceedingly well done and as complete as can be found in the higher-priced voluminous year-books. The value of the Röntgen rays as an aid in diagnosis is nicely illustrated in some of the reviews. The chapters on sanitary science, new instruments and new books cover the respective fields fairly well and add to the value of the work.

At the price for which it is sold, no physician can afford to be without the annual. The typography is similar in appearance and quality to the preceding volumes. W. C. K.

